# Seroepidemiology of HBV infection among health-care workers in South Sulawesi, Indonesia

**DOI:** 10.1186/s12879-018-3190-x

**Published:** 2018-06-18

**Authors:** Teguh Wijayadi, Rizalinda Sjahril, Turyadi, Susan I. Ie, Ridha Wahyuni, Ilhamjaya Pattelongi, M. Nasrum Massi, Irawan Yusuf, David H Muljono

**Affiliations:** 1 Universitas Hasanuddin, Makassar, Indonesia; 2 Tarumanegara University, Jakarta, Indonesia; 3 Eijkman Institute for Molecular Biology, Jakarta, Indonesia; 4 Faculty of Medicine and Health, University of Sydney, Camperdown, Australia

**Keywords:** Hepatitis B, Hepatitis B virus, Health-care workers, Sulawesi, Indonesia

## Abstract

**Background:**

Hepatitis B virus (HBV) infection is a world health problem with an estimated 257 million chronically infected people. Indonesia, with 7.1% prevalence of hepatitis B surface antigen (HBsAg), is classified as a moderately endemic country. Healthcare workers (HCWs) are at high occupational risk for HBV infection and potentially becoming transmitters for further infections. In Indonesia, the extent of hepatitis B among HCWs and specific control strategy are not available. This study evaluated the seroprevalence of HBV infection and associated risk factors in HCWs from four areas in South Sulawesi, Indonesia.

**Methods:**

A total of 467 HCWs (median age 28 years, male/female 89/378) were recruited. All HCWs were classified into three age groups (< 20–29, 30–39, and ≥ 40 years old), three work types (administration, non-intervention, and intervention), and three service periods (< 5, 5–9, and ≥ 10 years). Data on socio-demographic characteristics and risk factors were obtained by questionnaire and serum samples were tested for HBV markers (HBsAg, its antibody [anti-HBs], and antibody to core antigen [anti-HBc]. Chi-square or Fisher’s exact test was used to determine differences in categorical variables, while risk factors were reported as odds ratios (OR).

**Results:**

The prevalence of current HBV infection (HBsAg+), exposure to HBV (anti-HBc+), and immunity to HBV (anti-HBs+) was 6.2, 19.2, and 26.1%, respectively. Two thirds (66.17%) of all HCWs did not express any of HBV markers. In relation to the age groups, intervention work-type, and service period of HCWs, increasing trends were observed in the exposure to HBV (*p* < 0.001, *p* < 0.001, and *p* < 0.010, respectively) and the immunity to HBV by natural infection (HBsAg-, anti-HBc+, anti-HBs+) (*p* = 0.004, *p* < 0.001, and *p* < 0.010, respectively). Needlestick injury contributed the highest risk factor (OR = 1.71; 95% CI: 1.05–2.77; *p* = 0.029) for infection acquisition, which mostly occurred in the intervention group (*p* = 0.046).

**Conclusion:**

Exposure to HBV showed significant association with HCWs’ age, work type, and service period. Needlestick injury was the highest risk factor for the acquisition of HBV, with highest events in the intervention work-type. Two thirds of HCWs were still susceptible to HBV infection. Intervention strategies at the national level are required to mount prevention, control, and management of HBV infection in HCWs.

**Electronic supplementary material:**

The online version of this article (10.1186/s12879-018-3190-x) contains supplementary material, which is available to authorized users.

## Background

Hepatitis B virus (HBV) infection is one of the world infectious problems with an estimated 257 million chronically infected people. This infection accounts for 887,000 deaths annually due to its complications, including cirrhosis and hepatocellular carcinoma [1]. Indonesia with a total population of more than 250 million people has 7.1% prevalence of hepatitis surface antigen (HBsAg), and therefore is classified as a moderately hepatitis B endemic country [2].

Several groups have been assigned as special populations who have particular risks for acquiring HBV infection. Among these are the health-care workers (HCWs) who have occupational hazard of getting infected by this virus from their work place; and correspondingly, HBV-infected HCWs may potentially transmit HBV to patients and their families [3]. It has been shown that HCWs have an up to four-fold incidence of this infection in the general population. The main risk factor to acquire HBV infection for HCWs is direct contact with infectious material, especially HBV-infected blood or body fluid. Some studies also have reported that awareness of HBV and proper precautions against blood-borne infections are lacking in these workers [4].

Preventive vaccination as part of occupational safety measures has been standard in many countries. Nevertheless, it is still not formulated in many resource-poor settings [4, 5]. The World Health Organization (WHO) has reported that HBV vaccination coverage among HCWs is only 18–39% in low- and middle-income countries (LMIC) in comparison to 67–79% in high-income countries. This could be a consequence of lack of formulated policy and guidelines for the prevention and control of HBV infection among HCWs in most LMICs [6–8].

Recognizing this particular problem, the Global Health Sector Strategy (GHSS) on Viral Hepatitis 2016–2020 that was adopted by the World Health Assembly in May 2016 has stated occupational health measures as the core intervention and priority actions for countries to combat viral hepatitis [9]. More recently, the WHO Regional Office for South-East Asia in July 2017 has included routine hepatitis B vaccination among HCWs as a strategic direction in the Regional Action Plan for Viral Hepatitis 2016–2021 [10]. In Indonesia, efforts to control viral hepatitis have been made and supported with increasing commitment by the government [11]. However, the extent of hepatitis B among HCWs is unknown and specific strategy to control this problem has not been in place.

Over the course of more than 40 years since the first published cases of HCW-to-patient HBV transmission, there have been numerous publications on HBV infection among HCWs covering areas of epidemiology and intervention strategies [5, 12]. Most of the publications came from countries with low endemicity. Notably, there is still paucity of information on this particular problem in Indonesia [13, 14]; although such information is urgently needed to know the magnitude of the problem and to have accurate information on epidemiological estimates of the disease burden. This preliminary study strived to present information of HBV infection among HCWs by evaluating the seroprevalence of HBV infection and associated risk factors in health workers four areas in South Sulawesi Province, an area of transitional zone between the western part of Indonesia that have better social-economics and lower HBV infection, and the eastern part with less development and higher HBV infection rates.

## Methods

### Study population

**Fig. 1:**
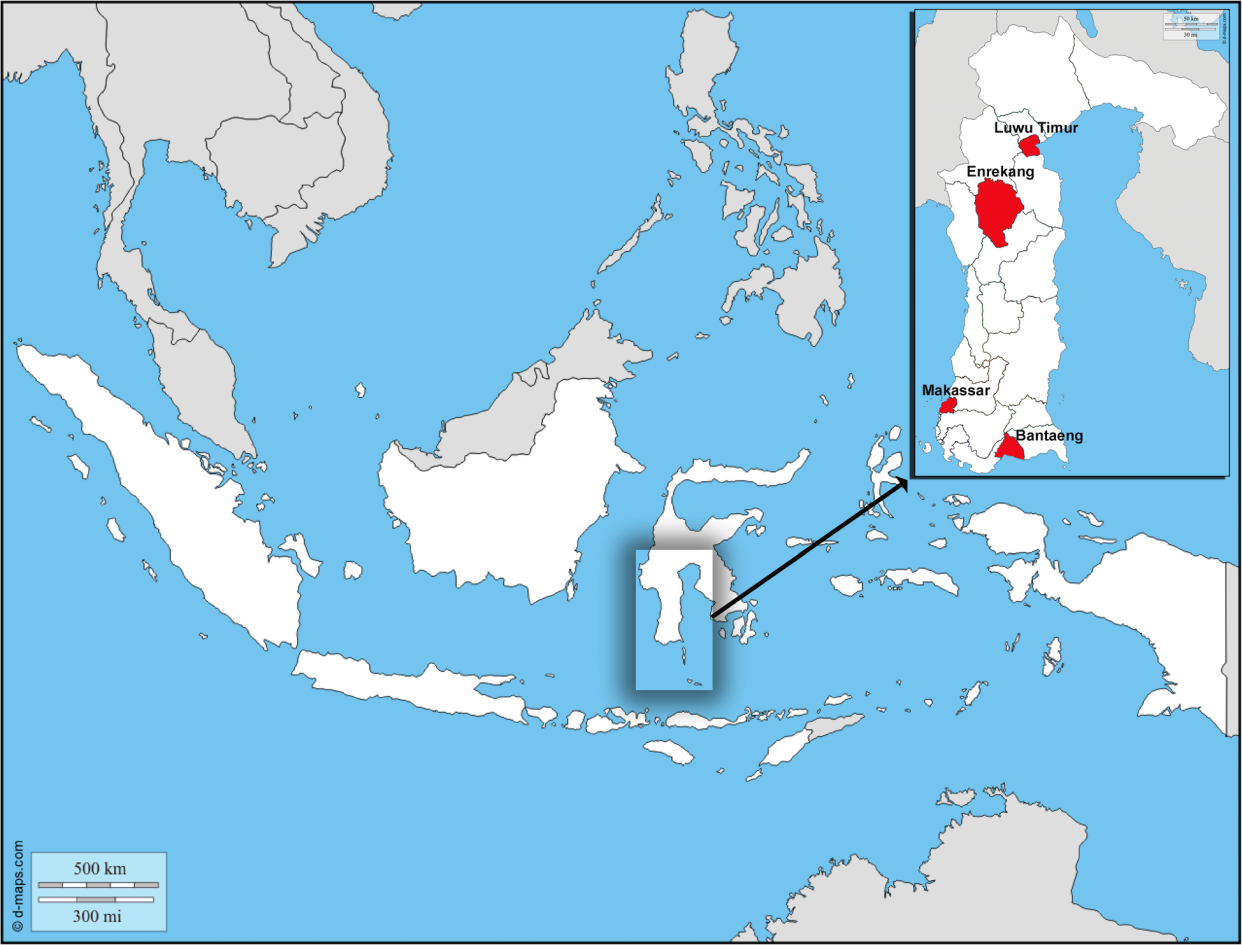
The position of studied areas (Luwu Timur, Enrekang, Makassar, and Bantaeng) of South Sulawesi, Province, Indonesia. The Island of Sulawesi is located in the transitional biogeographical zone between the western and eastern part of the Indonesian archipelago. (This figure is drawn based on the map outline obtained from: http://d-maps.com/carte.php?num_car=5487&lang=en; the insert is added to show the study site)

This study was conducted from June 2015 to October 2016 among HCWs from four areas (Makassar, Luwu Timur, Bantaeng, and Enrekang) of South Sulawesi Province, Indonesia (Fig. 1). A total of 467 HCWs, median age 28 (range 16–60) years, male/female 89/378, were involved in this study. After individual informed consent was obtained, a structured questionnaire was used to collect information on demographics, profession, duration in service, history of exposure to patient’s blood or body fluids, medical history, hepatitis B vaccination status, as well as risky behaviors such as intravenous drug user [4, 15, 16]. Serum samples were collected and stored at − 30 °C until use.

The HCWs were categorized into three groups by 10-year intervals; subjects of < 20–29 year-old were those from the youngest to 29.99 years, 30–39 year-old were from 30 to 39.99 years, and ≥ 40 year-old were from 40 years to the oldest. By type of work, the HCWs were categorized into: 1) Administration group, comprising administration and technical service staff; 2) Non-intervention group, comprising doctors, nurses and other personnel who were not exposed to materials contaminated with patient’s blood or body fluid in their routine work, such as ophthalmologists, dermatologists, psychiatrists, and allied health service personnel; 3) Intervention group, comprising HCWs with exposure-prone procedures, such as surgeons, gynecologists, midwives, dentists, laboratory staffs, and cleaning personnel [3, 4, 16].

All participants were considered as healthy asymptomatic subjects with no recorded clinical signs and symptoms related to liver diseases. All subjects were coded based on the institute’s sample numbering system to ensure the confidentiality. The original data were kept separately by the principal investigator and made inaccessible by other people including the researchers. The principle investigator reported the results of the examination in sealed envelopes to individual subjects. Subjects who tested positive for either HBsAg or anti-HBc were given referral letters to the nearest health-care facilities for further follow-up. All identities of subjects were discarded once the study was finished.

### Laboratory investigation

The serological markers of HBV tested were HBsAg, antibody to HBsAg (anti-HBs), and total immunoglobulin IgM and IgG antibodies to hepatitis B core antigen (anti-HBc). A positive HBsAg result signifies current HBV infection, either acute or chronic. Presence of anti-HBc indicates the evidence of current, recent, or past infection, hereafter referred to as ‘exposure’. Anti-HBs is produced in response to HBsAg and confer immunity to re-infection and its presence indicates immunity to HBV infection following an infection or successful immunization with hepatitis B vaccine [17].

Serum samples were tested for HBsAg, anti-HBs, and anti-HBc using commercially available immunoassays (Monolisa HBsAg Ultra—sensitivity 0.08 ng/mL, Monolisa Anti-HBs Plus, and anti-HBc plus, respectively; Biorad Laboratories, France) according to the manufacturer’s instruction. Anti-HBs level ≥ 10 IU/l was considered positive [18, 19].

### Statistical analysis

The baseline data were summarized descriptively. Chi-square or Fisher’s exact test was used to determine the differences in categorical variables. Linear-by-linear association chi-square was used to assess the trend of HBsAg, anti-HBc, and anti-HBs prevalence across age group, type of work, and duration of service of the HCWs [4, 16]. Risk factors associated with transmission of HBV were analysed, and outcomes were reported as odds ratios (OR) with 95% confidence intervals (CI). A *p*-value of < 0.05 was considered significant. All statistical analyses were two-sided and performed using the Statistical Program for Social Sciences (IBM SPSS 22.0 for Windows; SPSS, IL, USA).

## Results

### Subject characteristics

Based on age of HCWs, three groups were defined: < 20–29 years (300/64.2%), 30–39 years (127/27.2%), and ≥ 40 years (40/8.6%). The HCWs were categorized according to the type of work into administration (89/19.1%), non-intervention (297/63.6%), and intervention (81/17.3%) groups, and stratified based on length of service periods < 5 years (271/58.0%), 5–9 years (155/33.2%), and ≥ 10 years (41/8.8%). No significant difference in the length of service periods between males and females (5.4 ± 6.0 years vs 4.8 ± 4.6 years; *p* = 0.22). A female predominance was observed in all types of work (*p* < 0.001) (Additional file 1).

### Serological profiles of HBV infection

The prevalence of current HBV infection (HBsAg+) was 6.2% (29/467), which was found in 7.9% (7/89), 4.7% (14/297), and 9.9% (8/81) of the administration, non-intervention, and intervention HCWs, respectively, with no statistical difference between these groups (*p* = 0.181). No statistical difference was observed between males and females.

Evidence of exposure to HBV (anti-HBc +) was detected in 19.2% (89/467), which was found in 13.5% (12/89), 16.5% (49/297), and 34.6% (28/81) of the administration, non-intervention, and intervention HCWs, respectively, with significant increasing trend across the three groups (*p* < 0.001). Of all HCWs, isolated anti-HBc was found in 2.1% (10/467).

Immunity against HBV infection (anti-HBs+) was observed in 122 (26.1%) HCWs, consisting of 54 (11.6%) subjects with ‘vaccinated’ or ‘isolated-anti-HBs’ pattern (HBsAg-, anti-HBc-, anti-HBs +), and 68 (14.6%) subjects with ‘resolved-infection’ or ‘non-isolated anti-HBs’ pattern (HBsAg-, anti-HBc+, anti-HBs+). A serological pattern considered as healing HBV infection (HBsAg+, anti-HBc+, anti-HBs+) was found in two subjects (0.4%) (Table 1).

**Table 1:** Serological profile of HBV infection among health-care workers in South Sulawesi, Indonesia

HBV Serological parameter			Interpretation	Health care workers (HCWs)^a^				*P*-value
HBsAg	Anti-HBc	Anti-HBs		Overall N (%)	Administration n (%)	Non-intervention^b^n (%)	Intervention^c^ n (%)	
+	+	+	Chronic infection (considered as healing infection)	2 (0.43)	–	2 (0.7)	–	
+	+	–	Chronic infection	12 (2.57)	6 (6.7)	6 (2.0)	–	
+	–	+	Possible HBsAg mutant	1 (0.21)	–	1 (0.3)	–	
+	–	–	Chronic infection (isolated HBsAg)	14 (3.00)	1 (1.1)	5 (1.7)	8 (9.9)	< 0.001
–	+	+	Resolved from infection (naturally acquired immunity)	65 (13.92)	5 (5.6)	36 (12.1)	24 (29.6)	< 0.001
–	+	–	Isolated anti-HBc (possible HBsAg mutant or occult infection)	10 (2.14)	1 (1.1)	5 (1.7)	4 (4.9)	0.153
–	–	+	Isolated anti-HBs (immune due to vaccination)	54 (11.56)	6 (6.7)	38 (12.8)	10 (12.3)	0.285
–	–	–	Susceptible to infection	309 (66.17)	70 (78.7)	204 (68.7)	35 (43.2)	< 0.001
Total				467 (100.0)	89 (100.0)	297 (100.0)	81 (100.0)	

*HBV* hepatitis B virus, *HBsAg* hepatitis B surface antigen, *anti-HBc* antibody against HBV core antigen, *anti-HBs* antibody against HBV surface antigen

^a^The number of samples among the study population (overall and each work type) according to the serological parameter together with its percentage

^b^HCWs who were not exposed to materials contaminated with patient’s blood or body fluid in their routine work (e.g. ophthalmologists, dermatologists, psychiatrists, and allied health service personnel)

^c^HCWs with exposure-prone procedures to HBV-related infectious materials (e.g. surgeons, gynecologists, midwives, dentists, laboratory staffs, and cleaning personnel)

There were 309 (66.17%) HCWs who did not have any of HBV markers (HBsAg-, anti-HBc-, anti-HBs-) and thus classified as susceptible to infection. Based on type of work, they were found in 78.7% (70/89), 68.7% (204/297), and 43.2% (35/81) of the administration, non-intervention, and intervention groups, respectively. This distribution showed a downward trend from administration to non-intervention and intervention groups in susceptibility to HBV infection (*p* < 0.001).

### HBV infection status by demographic and occupational characteristics

HBV infection status with regard to gender, age, type of work, and length of service of the HCWs was assessed based on seropositive rates of HBsAg as a marker of current infection, anti-HBc as evidence of current or past exposure to HBV, and ‘infection-resolved’ or ‘non-isolated’ anti-HBs as a marker of natural boosting by repeated infection. Anti-HBc was significantly higher in males than females (*p* = 0.016), while other serological parameters were comparable between both genders.

**Fig. 2:**
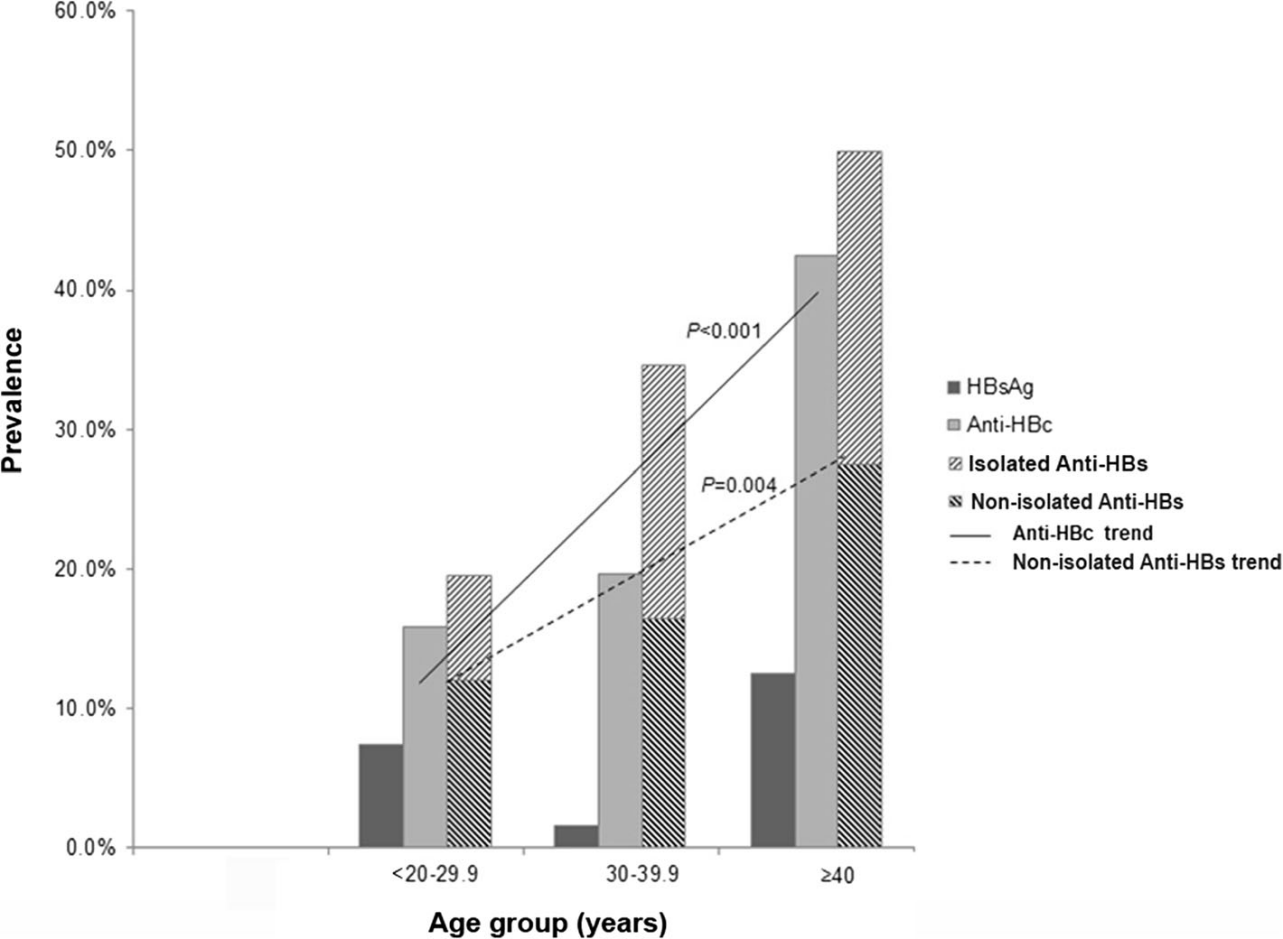
The prevalence of HBsAg, anti-HBc, and anti-HBs according to the age groups (< 20, 20–29.9, 30–39.9, and ≥ 40 years) among 467 health-care workers (HCWs). Linear-by-linear association test showed significant increasing trends of anti-HBc (*p* < 0.001) and non-isolated anti-HBs (*P* = 0.004). No significant difference was found in the prevalence of vaccinated-anti-HBs among the three groups

The prevalence HBsAg did not show significant difference in the three (20–29, 30–39, and ≥ 40 years) age groups. However, the prevalence of anti-HBc among the three groups was 15.7% (47/300), 19.7% (25/127), and 42.5% (17/40), respectively (*p <* 0.001), while that of non-isolated anti-HBs was 12.0% (36/300), 16.5% (21/127), and 27.5% (11/40), respectively (*p =* 0.004). This finding showed rising trends of the two markers as age increased, indicating accumulated exposure to HBV infection by increasing age (Fig. 2).

**Fig. 3:**
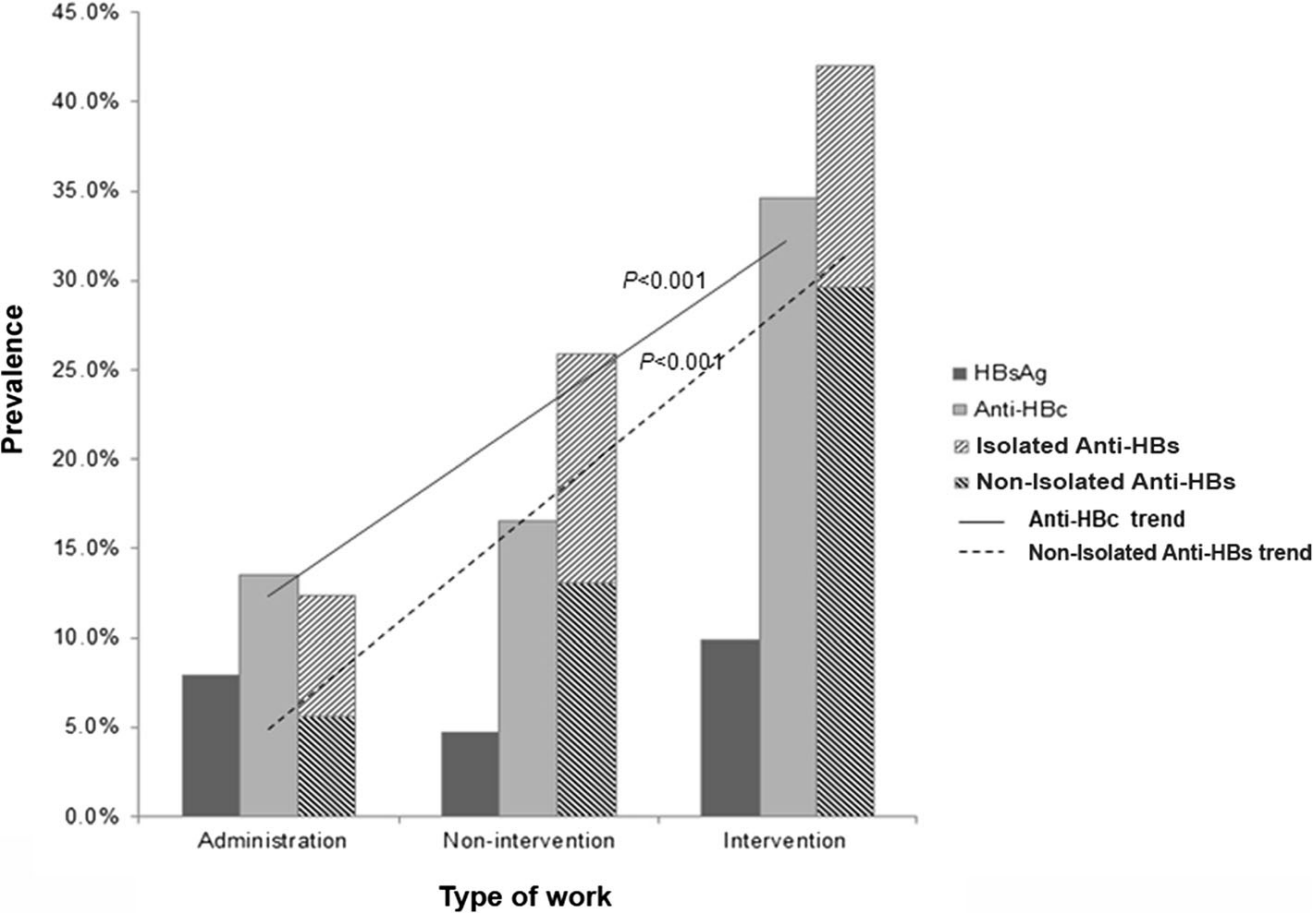
The prevalence of HBsAg, anti-HBc, and anti-HBs in the administration, non-intervention, and intervention groups among 467 health-care workers (HCWs). Linear-by-linear association test showed significant increasing trends of anti-HBc (*p* < 0.001) and non-isolated anti-HBs (*p* < 0.001). No significant difference was found in the prevalence of vaccinated-anti-HBs among the three groups

Based on type of work (Fig. 3), the prevalence of HBsAg in the administration, non-intervention, and intervention groups was 7.9% (7/89), 4.7% (14/297), and 9.9% (8/81), respectively, with no significant difference between the groups. The prevalence of anti-HBc in the three groups was 13.5% (12/89), 16.5% (49/297), and 34.6% (28/81), respectively (*p* < 0.001), while that of non-isolated anti-HBs was 5.6% (5/89), 13.1% (39/297), and 29.6% (24/81), respectively (*p* < 0.001).

**Fig. 4:**
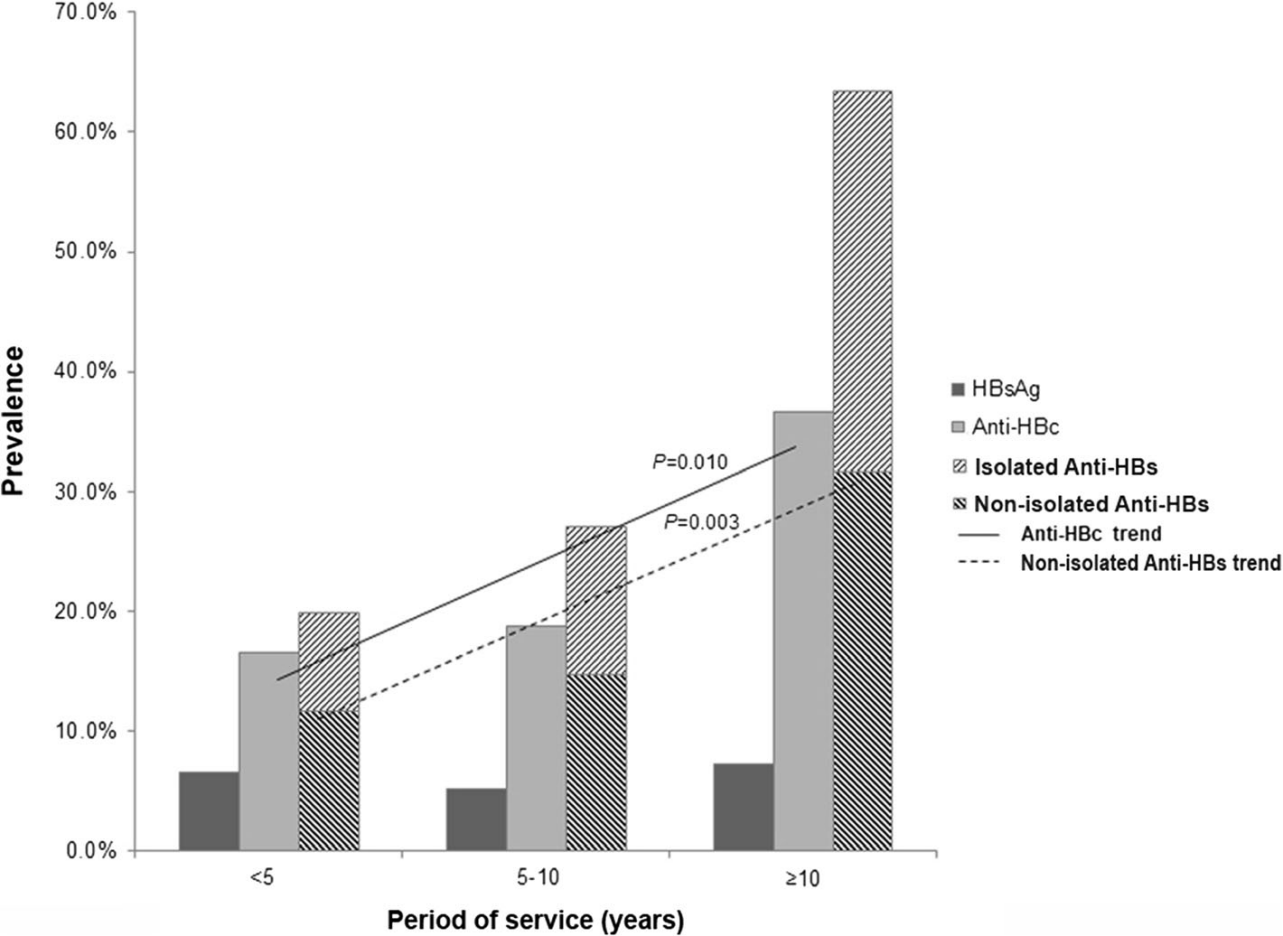
The prevalence of HBsAg, anti-HBc, and anti-HBs based on the length of service period (< 5, 5–10, and ≥ 10 years) among 467 health-care workers (HCWs). Linear-by-linear association test showed significant increasing trends of anti-HBc (*p* = 0.010) and anti-HBs and non-isolated anti-HBs (*p* = 0.003). No significant difference was found in the prevalence of vaccinated-anti-HBs among the three groups

When analyzed by length of service (Fig. 4), the prevalence of HBsAg for < 5, 5–9, ≥10 service years was 6.6% (18/271), 5.2% (8/155), and 7.3% (3/41), respectively, with no significant difference between the groups. The prevalence of anti-HBc in the three service-year groups was 16.6% (45/271), 18.7% (29/155), and 36.6% (15/41), respectively (*p* < 0.010), while that of non-isolated anti-HBs was 11.8% (32/271), 14.8% (23/195), and 31.7% (13/41), respectively (*p* = 0.003). These findings showed escalating trends of HBV infection across the three types of work and the length of service period of HCWs.

### Risk factors for current infection and exposure to hepatitis B virus

#### Risk factors for current HBV infection

As shown in Table 2, no significant difference was observed in the prevalence of current HBV infection for most variables, except for the age that was associated with a higher risk in HCWs aged ≥40 years than those in 30–39 year-old group (OR 8.93; 95% CI 1.66–48.01; *p* = 0.009).

**Table 2:** Risk factors for current and exposure to HBV infection among health care workers [4, 15, 16]

Variable	Overall	Current HBV infection			Exposure to HBV infection		
	(*N* = 467)	HBsAg+ (%)^a^	OR (IC 95)	*p-*value^b^	Anti-HBc + (%)^a^	OR (IC 95)	*p-*value^b^
Gender							
Male	89	7.9	1.38 (0.57–3.34)	0.412	28.1	1.92 (1.12–3.27)	**0.016**
Female (Ref)	378	5.8	1		16.9	1	
Age group (Years)							
< 20–29 (Ref)	300	7.3	1		15.7	1	
30–39	127	1.6^d^	0.20 (0.47–0.87)	**0.018**	19.7^e^	1.32 (0.77–2.26)	0.311
≥ 40	40	12.5^d^	1.81 (0.64–5.07)	0.344^c^	42.5^e^	3.98 (1. 98–8.01)	**< 0.001**
Marital status							
Married/Separated	277	6.9	1.31 (0.60–2.89)	0.500	21.7	1.52 (0.93–2.47)	0.093
Single (Ref)	188	5.3	1		21.54	1	
Type of Work							
Administration (Ref)	89	7.9	1		13.5	1	
Non-intervention	297	4.7	0.58 (0.23–1.48)	0.250	16.5^f^	1.27 (0.64–2.51)	0.494
Intervention	81	9.9	1.28 (0.44–3.71)	0.644	34.6^f^	3.39 (1.58–7.26)	**< 0.001**
Length of service (years)							
< 5 (Ref)	271	16.6	1		16.6	1	
5–9	155	5.2	0.76 (0.33–1.80)	0.539	18.7^g^	1.16 (0.69–1.93)	0.581
≥ 10	41	7.3	1.11 (0.31–3.95)	0.872	36.6^g^	2.90 (1.42–5.90)	**0.002**
Needlestick injury							
Yes	160	4.4	0.53 (0.22–1.27)	0.149	24.4	1.71 (1.05–2.77)	**0.029**
No (Ref)	277	7.9	1		15.9	1	
Other work-related injuries							
Yes	41	0.0	0.00 (−)	0.580^c^	26.8	1.55 (0.73–3.28)	0.252
No (Ref)	282	29.2	1		19.1	1	
Hepatitis B vaccination history							
Unvaccinated/unknown	322	7.5	1.12 (0.33–3.91)	0.850	18.9	1.87 (0.71–4.94)	0.200
Vaccinated (Ref)	45	6.7	1		11.1	1	
Blood recipient							
Yes	11	0.0	0.00 (−)	1.000^c^	27.3	1.59 (0.42–6.11)	0.498^c^
No (Ref)	445	6.5	1		19.1	1	
History of jaundice							
Yes	21	9.5	1.59 (0.35–7.17)	0.636	28.8	1.69 (0.64–4.49)	0.286
No (Ref)	434	6.2	1		19.1	1	
Family history of liver disease							
Yes	30	6.7	1.06 (0.24–4.67)	1.000^c^	30.0	1.91 (0.84–4.35)	0.115
No (Ref)	410	6.3	1		18.3	1	

*OR* odds ratio, *CI* confidence interval

^a^The percentage of samples in each variable

^b^Chi square test, except

^c^by Fisher exact test; significant *p* values (< 0.05) are in bold

^d^Risk of current HBV infection for age group ≥40 y.o. vs 30–39 y.o: OR 8.93 (95% CI, 1.66–48.01; *p* = 0.009^3^)

^e^Risk of exposure to HBV infection for age group ≥40 y.o. vs 30–39 y.o: OR 3.02 (95% CI: 1.40–6.48; *p* = 0.004)

^f^Risk of exposure to HBV infection for type of work Intervention vs non-intervention: OR 2.67 (95% CI: 1.54–4.64; *p* < 0.001)

^g^Risk of exposure to HBV infection for length of service ≥10 years vs 5–9 years: OR = 2.51 (95% CI: 1.18–5.32; *p* = 0.015)

#### Risk factors for exposure to HBV infection

Males had a significantly higher risk of contracting HBV (OR 1.92; 95% CI: 1.12–3.27; *p* = 0.016). HCWs of ≥40 years old were at higher risk for acquiring HBV infection than those in the < 20–29 year-old groups (OR 3.98; 95% CI: *p* < 0.001) and 30–39 (OR 3.02; 95% CI: 1.40–6.48; *p* = 0.009). No significant difference was found between 20 and 29 and 30–39 year-old groups.

By job category, the intervention group had significantly a higher risk of acquiring HBV infection compared to the administration (OR 3.39; 95% CI: 1.58–7.26; *p* = 0.001) and non-intervention groups (OR 2.67; 95% CI: 1.54–4.64; *p* < 0.001). No significant difference in acquisition risk of HBV infection was found between administration and non-intervention groups.

Further investigation was made to identify the contribution of the length of employment to the risk for getting infected with HBV. Having been working for ≥10 years was associated with a higher risk for acquisition of HBV infection compared to those who had been in service for < 5 years (OR 2.90; 1.42–5.90; *p* = 0.002) and 5–9 years (OR 2.51; 95% CI: 1.18–5.32; *p* = 0.015). No risk difference was found between those who had working duration of < 5 and 5–9 years.

Among occupational factors, needlestick injury contributed a higher risk (OR = 1.71; 95% CI: 1.05–2.77; *p* = 0.029) for the acquisition of HBV infection. The highest events of needlestick injury were experienced by the intervention group (Additional file 1: Table S1) in comparison to their counterparts (*p* = 0.046).

### Vaccination against HBV infection

Of 367 HCWs who answered the question on vaccination status, 45 (12.3%) stated that they had been vaccinated against HBV, with 21 (6.1%) completed the three dose schedule, 10 (3.1%) had two vaccinations, and 14 (3.3%) had one vaccination (Additional file 1). The others had never received hepatitis B vaccination or did not remember whether they had been vaccinated, because the national infant hepatitis B immunization had not been introduced during their childhood. No significant difference was found in the prevalence of current infection and exposure rates between the vaccinated and unvaccinated groups. When serologically tested, anti-HBs positive results were found in 28 (62.2%) among the 45 vaccinated subjects, while HBsAg was positive in 3 (6.7%) and anti-HBc was positive in 5 (11.1%) (Additional file 2). The rest variables with potential risks were not significantly associated with current infection or exposure to HBV infection.

## Discussion

This study revealed a high burden of HBV infection among HCWs from four areas in South Sulawesi Province of Indonesia. The overall prevalence of current HBV infection (HBsAg positivity) was 6.2%, and that for exposure to HBV infection (anti-HBc positivity) was 19.2%. These figures were lower than the average national prevalence of HBsAg and anti-HBc (7.1 and 31.9%, respectively) and that in the same province (7.6 and 39.4%, respectively) as released by the National Health Survey in 2013 [2, 20, 21]. Immunity to HBV infection was present in 26.1% of HCWs, which was attained through resolved infection in 14.6% subjects. The proportion of subjects who had immunity due to natural infection provides further evidence that HCWs have a high risk of becoming infected with HBV through their occupation [4, 16, 22]. Of all HCWs, 66.7% were still susceptible to HBV infection.

A significantly higher rate of HBV exposure was observed in older HCWs than the younger ones. One explanation could be that there is a relatively constant risk of exposure during their service period time which results in the increase of hepatitis B prevalence with age [15]. This could be an additional risk to the existing phenomenon of horizontal HBV transmission as shown by the age-related increase of anti-HBc in the general population in the 2013 National Health Survey [2, 21].

As an important occupational hazard, the work category of HCW may reflect the varying levels of risk of exposure to a HBV infection. To the best of our knowledge, this is the first published study based on job category of HCWs in Indonesia which includes serological analysis and risk calculation with confidence intervals. Our findings demonstrated the increasing exposure and immunity rates to HBV across the three types of work: administration, non-intervention, and intervention. This result is consistent with the pattern observed in other countries, regardless of economic status and hepatitis B endemicity, that HCWs who perform invasive procedures have been consistently shown to have higher rates of HBV infection than their counterparts [4, 5, 16].

In this study, longer duration in service did not give significant contribution to the risk of current infection. This result is in line with other reports from hepatitis B endemic areas, with an assumption that the prevalence of hepatitis B in HCWs could be as high as in the general population [4]. On the other hand, this study showed that long occupational period was associated with higher exposure rates and natural immunity to HBV infection. This finding is consistent with other studies [4, 23, 24]; however, it could be associated with the higher acquisition of HBV markers which also increase by age [16].

In line with other studies, the most common mode of exposure to HBV infection was through needlestick which occurred mostly in the intervention group [4, 7, 13–16]. Even though HCWs have repeated a given procedure so many times, one slip can cause injury with potentially serious consequences. An unexpected or sudden movement by the patient or a transient lack of concentration can result injury [22, 25, 26]. Other possible reasons for high prevalence of needlestick injuries include lack of specific measures to address occupational challenges, lack of information, and non-adherence to standard precautions [26]. Adverse schedule characteristics such as long work hours can result in stress, emotional and physical exhaustion may also increase the chance of human error and poor compliance with the general precautions [5, 26].

In response to the risk of exposure, safeguards have been put in place to lessen the risk of injury. These include the adoption of universal precautions, needleless systems to connect with intravenous tubing, double gloving, and having a neutral zone in which to pass sharp instruments, use of puncture resistant, leak proof, and labelled or color-coded containers [25, 27]. In addition, devices have been developed to reduce injuries including retractable needles and syringes with a sliding sheath [28]. Guidelines have been developed for the management of exposure to HBV that involves proper risk assessment, determination of HBV status of the source and the exposed, and the administration of post-exposure prophylaxis as appropriate [29]. Hepatitis B immunoglobulin can be offered for immediate protection upon significant exposure to HBV, while HBV vaccination can be given to individuals who lack HBsAg and have not been vaccinated or developed satisfactory immune response after previous completed immunization series [17, 30].

Among 367 questionnaire respondents, only 45 (12.3%) HCWs had been vaccinated against HBV. This vaccination coverage is very low compared to developing countries like India, Pakistan, and several countries in Africa [8, 16, 31, 32]. Within this vaccinated group, immunity to HBV could be demonstrated in 62.2%, while 37.2% were in doubtful protection despite feeling well protected. Indeed, of the vaccinated subjects, HBsAg was positive in 6.7% and anti-HBc was positive in 11.1%.

Indonesia has implemented universal hepatitis B vaccination to all infants since 1997 and administered birth dose immunization since 2000. However, nationwide hepatitis B vaccination program for high risk groups including HCWs has not been in place. In hepatitis B endemic countries where people have high rates of natural immunity, providing universal HBV vaccination for HCWs is often discussed because of questionable cost effectiveness of this preventive measure [33]. Nevertheless, this present study showed that 66.17% of HCWs did not have any of HBV serological markers, and therefore susceptible and at risk of acquiring HBV infection.

Considering the high exposure rates to HBV revealed in this study, these subjects highly need vaccination to be protected. This is in line with the recent WHO updated position paper on hepatitis B vaccine – July 2017, which recommends that HCWs and other groups with occupational exposure should be the targets for vaccination [34]. It is emphasized that hepatitis B vaccination safeguards health workers when administered early, ideally before occupational exposure. Drawing upon the GHSS on Viral Hepatitis 2016–2020 and the WHO Regional Action Plan for Viral Hepatitis in South-East Asia 2016–2021, it is targeted that all Member States have started implementation of routine hepatitis B vaccination among high-risk groups including health-care workers by 2020 [9, 10].

In some resource-limited countries, approaches have been made to avoid unnecessary vaccinations. Introduction of point-of-care tests for HBsAg and anti-HBs in a pre-vaccination screening of HCWs could be cost-effective by not giving unnecessary vaccinations for individuals already infected, save vaccine for those having immunity to HBV, and provide an opportunity to refer people with HBV infection for care and treatment [4]. Although routine post-vaccination testing is not recommended, persons at risk of occupational exposure to HBV infection including HCWs should be considered for post-vaccination testing where laboratory facilities are available to ensure that they have achieved a protective anti-HBs level (≥10 mIU/mL) [17, 34].

We acknowledge several limitations of this study. Firstly, the cross-sectional design that did not make it possible to accurately detect serological HBV markers that might wean or fluctuate over times at low titers, which could be different than the rates revealed in this study. Secondly, this study could not accurately define the job changes of some HCWs that might happen in certain individuals due to age or physical reasons. However, it should not be a major problem as job changes were not frequent occurrences; job assignments were based on the skills of HCWs that would need certain qualification to perform a given duty. Thirdly, the coverage of the study that investigated health workers from four areas of South Sulawesi Province could not represent the large HCW population of the Indonesian archipelago. Similar studies in areas with different socio-economic development, lifestyle, and prevalence of HBV infection would be needed. However, by performing serological analysis in each work type and employment periods, combined with risk calculation of the HCWs, this study provides the evidence of increasing exposure to HBV infection associated with job categories and length of employment among the HCWs.

## Conclusions

This study reported the occupational risk of acquiring HBV infection among the studied HCW population. The risk is associated with the age, type of work, and length of service of HCWs, particularly in the profession that routinely perform exposure-prone procedures. Needle-stick injury contributed the highest risk among the variables investigated. A particular attention has to be given to two-thirds of HCWs who were still susceptible to HBV infection. These facts confirm the necessity to safeguard the HCWs with hepatitis B vaccination, which further provides greater protection to patients from infection through exposure to infected health workers. Infection control needs to be strengthened and continuing education has to be imparted to all HCWs at various health-care setups. A policy and roadmap for intervention at the national level is required to mount effective and efficient measures for the prevention, diagnosis, post-exposure management, and treatment of HBV infection in this special population.

## Additional files

Additional file 1: **Table S1**. Distribution of demographic variables and risk factors according to the type of work among health-care workers. (DOCX 26 kb)

Additional file 2: **Table S2**. Serological profile of HBV infection among health-care workers according to hepatitis B vaccination status. (DOCX 21 kb)

## Abbreviations

anti-HBc: Antibody to hepatitis B core antigen
anti-HBc: Antibody to hepatitis B surface antigen
CI: Confidence interval
GHSS: Global Health Sector Strategy
HBsAg: Hepatitis B surface antigen
HBV: Hepatitis B virus
HCW: Health-care worker
LMIC: Low- and middle-income countries
OR: Odds ratio
SPSS: Statistical Program for Social Sciences
WHO: World Health Organization
