# Supplementary material for: Seroepidemiology of HBV infection among health-care workers in South Sulawesi, Indonesia
Source: BMC Infect Dis. 2018 Jun 18;18:279. doi: 10.1186/s12879-018-3190-x (PMC6006550; doi:10.1186/s12879-018-3190-x)
Supplement: Supplementary file 1 — Table S1. Distribution of demographic variables and risk factors according to the type of work among health-care workers. (DOCX 26 kb) [file 12879_2018_3190_MOESM1_ESM.docx]

**Additional file 1: Table S1 Distribution of demographic variables and risk factors according to the type of work among health-care workers**

| Variable | Overall |  | Type of work^1^ | | | | | P value^3^ | |
| --- | --- | --- | --- | --- | --- | --- | --- | --- | --- |
|  |  |  | Administration |  | Non-intervention |  | Intervention |  |  |
|  | N (%) |  | n (%) |  | n (%) |  | n (%) |  |  |
| Gender |  |  |  |  |  |  |  |  |  |
| Male | 89 (19.1) |  | 21 (23.6) |  | 35 (11.8) |  | 33 (40.7) | **<0.001** |  |
| Female | 378 (80.9) |  | 68 (76.4) |  | 262 (88.2) |  | 48 (59.3) |  |  |
| Age group (Years) |  |  |  |  |  |  |  |  |  |
| <20-29 | 300 (64.2) |  | 48 (53.9) |  | 203 (68.4) |  | 49 (60.5) | **0.042** |  |
| 30-39 | 127 (27.2) |  | 28 (31.5) |  | 76 (25.6) |  | 23 (28.4) |  |  |
| ≥40 | 40 (8.6) |  | 13 (14.6) |  | 12 (6.1) |  | 9 (11.1) |  |  |
| Marital status^†^ |  |  |  |  |  |  |  |  |  |
| Single | 188 (40.4) |  | 29 (32.6) |  | 130 (44.1) |  | 29 (35.8) | 0.099 |  |
| Married/Separated | 277 (59.6) |  | 60 (67.4) |  | 165 (55.9) |  | 52 (64.2) |  |  |
| Length of service period (years) |  |  |  |  |  |  |  |  |  |
| <5 | 271 (58.0) |  | 60 (67.4) |  | 173 (58.2) |  | 38 (46.9) | 0.083 |  |
| 5-9 | 155 (33.2) |  | 25 (28.1) |  | 97 (32.7) |  | 33 (40.7) |  |  |
| ≥10 | 41 (8.8) |  | 4 (4.5) |  | 27 (9.1) |  | 10 (12.3) |  |  |
| Needle-stick injury^2^ |  |  |  |  |  |  |  |  |  |
| Yes | 160 (36.6) |  | 23 (26.1) |  | 104 (38.0) |  | 33 (44.0) | **0.046** |  |
| No | 277 (63.4) |  | 65 (73.9) |  | 170 (62.0) |  | 42 (56.0) |  |  |
| Other work-related injuries^2^ |  |  |  |  |  |  |  |  |  |
| Yes | 41 (12.7) |  | 5 (8.5) |  | 26 (13.1) |  | 10 (15.2) | 0.511 |  |
| No | 282 (87.3) |  | 54 (91.5) |  | 172 (86.9) |  | 56 (84.8) |  |  |
| Hepatitis B immunization history^2^ |  |  |  |  |  |  |  |  |  |
| Unvaccinated/unknown | 322 (87.7) |  | 75 (90.6) |  | 199 (86.9) |  | 48 (87.3) | 0.708 |  |
| Vaccinated | 45 (12.3) |  | 8 (9.6) |  | 30 (13.1) |  | 7 (12.7) |  |  |
| Blood recipient^2^ |  |  |  |  |  |  |  |  |  |
| Yes | 11 (2.4) |  | 0 (0.0) |  | 6 (2.1) |  | 5 (6.3) | **0.024** |  |
| No | 445 (97.6) |  | 89 (100) |  | 282 (97.7) |  | 74 (93.7) |  |  |
| History of jaundice^2^ |  |  |  |  |  |  |  |  |  |
| Yes | 21 (4.6) |  | 4 (4.5) |  | 9 (3.1) |  | 8 (9.9) | **0.039** |  |
| No | 434 (95.4) |  | 84 (95.5) |  | 277 (96.9) |  | 73 (90.1) |  |  |
| Family history of liver disease^2^ |  |  |  |  |  |  |  |  |  |
| Yes | 30 (6.8) |  | 2 (2.2) |  | 25 (9.0) |  | 3 (4.1) |  |  |
| No | 410 (93.2) |  | 87 (97.8) |  | 252 (91.0) |  | 71 (95.9) | 0.051 |  |

^1^Number of subjects in each type of work with its percentage; ^2^The number of answered questions in the questionnaire; ^3^Chi square or Fischer exact test; significant *p* values (<0.05) are in bold.

Supplementary Table 2. Serological profile of HBV infection among health-care workers according to hepatitis B vaccination status*

| Hepatitis B vaccination status | N | HBsAg+ | anti-HBc+ | Anti-HBs + |
| --- | --- | --- | --- | --- |
|  |  | n (%)† | n (%)† | n (%)† |
| Vaccinated | 45 | 3 (6.7) | 5 (11.1) | 28 (62.2) |
| Unvaccinated | 322 | 24 (7.5) | 61 (18.9) | 65 (20.2) |
| Total | 367 | 27 (7.4) | 66 (18.0) | 93 (25.3) |

*Of 367 questionnaire respondents; †The number positive result and its percentage according to vaccination status
