# Supplementary material for: Seroepidemiology of HBV infection among health-care workers in South Sulawesi, Indonesia
Source: BMC Infect Dis. 2018 Jun 18;18:279. doi: 10.1186/s12879-018-3190-x (PMC6006550; doi:10.1186/s12879-018-3190-x)
Supplement: Supplementary file 2 — Table S2. Serological profile of HBV infection among health-care workers according to hepatitis B vaccination status. (DOCX 21 kb) [file 12879_2018_3190_MOESM2_ESM.docx]

**Additional file 2: Table S2**. Serological profile of HBV infection among health-care workers according to hepatitis B vaccination status^1^

| Hepatitis B vaccination status | N | HBsAg+ | anti-HBc+ | Anti-HBs + |
| --- | --- | --- | --- | --- |
|  |  | n (%)^2^ | n (%)^2^ | n (%)^2^ |
| Vaccinated | 45 | 3 (6.7) | 5 (11.1) | 28 (62.2) |
| Unvaccinated | 322 | 24 (7.5) | 61 (18.9) | 65 (20.2) |
| Total | 367 | 27 (7.4) | 66 (18.0) | 93 (25.3) |

^1^Of 367 questionnaire respondents; ^2^The number positive result and its percentage according to vaccination status
